# Kaleidoscope heart

**Published:** 2019-03-13

**Authors:** Rosetta Mazzola

**Affiliations:** 1 University of British Columbia, British Columbia, Canada

**Figure:**
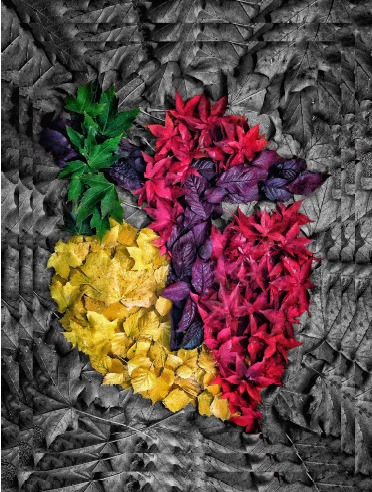


I am an enthusiastic amateur photographer, taking great pleasure in capturing landscapes but also dabbling in portrait and still life photography. This image was inspired by my daily bicycle commute to and from UBC's Point Grey campus during my first semester of medical school in the fall of 2016. Every day, I cycled through the ever-changing landscape of Vancouver. While we were studying cardiology, in my mind’s eye, the colourful leaves falling from the trees formed the chambers and vessels of a heart. This playful fantasy reminded me of the importance of maintaining an open mind in recognizing patterns when diagnosing patients in medicine.

## About the author

Rosetta Mazzola is a third-year medical student at the University of British Columbia’s Southern Medical Program (SMP) in Kelowna. She is planning to pursue a residency in internal medicine and is considering subsequently subspecializing, although her interests remain broad at this time. Prior to entering medical school, she completed her bachelor’s degree in biochemistry at the University of Victoria and worked for the Deeley Research Centre at the BC Cancer Agency. Outside of medicine, she enjoys being involved in student government as the SMP Site Lead to the Medical Undergraduate Society. In her free time, she likes to stay active and enjoys nature by cross-country skiing, rock climbing, and hiking – whether that be in her own back yard or while traveling abroad.

